# Rare-Earth Orthophosphates From Atomistic Simulations

**DOI:** 10.3389/fchem.2019.00197

**Published:** 2019-04-03

**Authors:** Yaqi Ji, Piotr M. Kowalski, Philip Kegler, Nina Huittinen, Nigel A. Marks, Victor L. Vinograd, Yulia Arinicheva, Stefan Neumeier, Dirk Bosbach

**Affiliations:** ^1^Forschungszentrum Jülich GmbH, Institute of Energy and Climate Research - IEK-6: Nuclear Waste Management and Reactor Safety, Jülich, Germany; ^2^JARA High-Performance Computing, Aachen, Germany; ^3^Institute of Resource Ecology, Helmholtz-Zentrum Dresden-Rossendorf, Dresden, Germany; ^4^Curtin University, Perth, WA, Australia; ^5^Forschungszentrum Jülich GmbH, Institute of Energy and Climate Research - IEK-1: Materials Synthesis and Processing, Jülich, Germany

**Keywords:** rare-earth phosphates, atomistic simulations, monazite, xenotime, nuclear waste management, ceramics, thermodynamics, solid solutions

## Abstract

Lanthanide phosphates (*LnPO*_4_) are considered as a potential nuclear waste form for immobilization of Pu and minor actinides (Np, Am, and Cm). In that respect, in the recent years we have applied advanced atomistic simulation methods to investigate various properties of these materials on the atomic scale. In particular, we computed several structural, thermochemical, thermodynamic and radiation damage related parameters. From a theoretical point of view, these materials turn out to be excellent systems for testing quantum mechanics-based computational methods for strongly correlated electronic systems. On the other hand, by conducting joint atomistic modeling and experimental research, we have been able to obtain enhanced understanding of the properties of lanthanide phosphates. Here we discuss joint initiatives directed at understanding the thermodynamically driven long-term performance of these materials, including long-term stability of solid solutions with actinides and studies of structural incorporation of *f* elements into these materials. In particular, we discuss the maximum load of Pu into the lanthanide-phosphate monazites. We also address the importance of our results for applications of lanthanide-phosphates beyond nuclear waste applications, in particular the monazite-xenotime systems in geothermometry. For this we have derived a state-of-the-art model of monazite-xenotime solubilities. Last but not least, we discuss the advantage of usage of atomistic simulations and the modern computational facilities for understanding of behavior of nuclear waste-related materials.

## 1. Introduction

Lanthanide phosphates (*LnPO*_4_) are ceramic materials of interest in various research fields, including geochronology (Williams et al., 2007), geothermometry (Andrehs and Heinrich, 1998; Mogilevsky, 2007), and nuclear waste management (Ewing and Wang, 2002; Neumeier et al., 2017a; Schlenz et al., 2017). Occurring in nature, these are also important ores for thorium, lanthanum, and cerium (McGill, 2000; Stoll, 2000). Most of the potential applications come from high durability and radiation damage resistance of these materials (Neumeier et al., 2017a). There exist large varieties of phosphate-based ceramics of different crystalline structures [e.g., cheralite, apatites, kosnarite, see Neumeier et al. (2017a)], but anhydrous lanthanide orthophosphates form two structures called monazite and xenotime. Anhydrous lanthanide orthophosphates crystallize in the monazite form with the *P*2_2_/*n* space group for light lanthanides, from La to Gd. In this structure, *Ln* cations are 9-fold coordinated. For heavier lanthanides, from Tb to Lu, *LnPO*_4_ compounds adopt a zircon type tetragonal structure called xenotime which has *I*4_1_/*amd* symmetry and 8-fold coordinated *Ln* cations. Both these materials are considered in nuclear waste management as potential immobilization matrices for minor actinides and plutonium because of enhanced radiation damage resistance and durability of these materials (Ewing and Wang, 2002; Clavier et al., 2011; Schlenz et al., 2013, 2017; Neumeier et al., 2017a; Seydoux-Guillaume et al., 2018). Their natural analogues can contain significant amounts of actinides [up to ~50%wt of Th and U, see Stoll (2000); Ewing and Wang (2002); Lumpkin and Geisler-Wierwille (2012)] and still preserve their crystalline structure over geological time scales (Ewing and Wang, 2002). The aim is a potential usage of these materials as an immobilization matrix for radionuclides, such as the already mentioned Pu and minor actinides. The immobilization of Pu could reduce the proliferation risk associated with large stockpiles of weapons grade plutonium (Ewing, 1999; Macfarlane, 1999).

In the last two decades, atomistic modeling became a very popular research tool in various research fields, including nuclear materials (Chroneos et al., 2013). This is because steady advancements in supercomputing facilities and computational software, especially *ab initio* methods, allows nowadays for simulation and investigation of systems containing hundreds of atoms using first principle simulation methods (Jahn and Kowalski, 2014). Monazite became a topic of atomistic simulations effort in the last decade. Computational studies have been used to deliver information on: the structural (Rustad, 2012; Feng et al., 2013; Blanca-Romero et al., 2014; Beridze et al., 2016; Huittinen et al., 2017), the elastic (Wang et al., 2005; Feng et al., 2013; Ali et al., 2016; Kowalski and Li, 2016; Ji et al., 2017a; Kowalski et al., 2017b), the thermodynamic (Mogilevsky, 2007; Feng et al., 2013; Li et al., 2014; Kowalski et al., 2015, 2016; Ji et al., 2017b; Neumeier et al., 2017b; Eremin et al., 2019), the thermochemical (Rustad, 2012; Beridze et al., 2016), the radiation damage resistance (Kowalski et al., 2016; Li et al., 2016; Ji et al., 2017c; Jolley et al., 2017) parameters, the electronic structure (Blanca-Romero et al., 2014; Kowalski et al., 2017a) as well as high-pressure behavior (López-Solano et al., 2010; Stavrou et al., 2012; Ali et al., 2016; Shein and Shalaeva, 2016; Gomis et al., 2017). There is a steadily increasing relevant simulation effort with most of the papers published just recently.

In this contribution we provide an overview of the recent atomistic modeling activities on the lanthanide-phosphates, focusing on the information that have been delivered by atomistic modeling activities at Forschungszentrum Jülich and that allowed for better characterization of these materials, including long-term thermodynamic stability in the context of using them as a nuclear waste form. Besides this overview, we present results of computation of thermochemical parameters of *La*_*1-x*_*Pu*_*x*_*PO*_4_ solid solution and the first *ab initio*-based evaluation of the relative solubilities in the monazite-xenotime system. These studies aim at the assessment of the maximum Pu load in *LaPO*_4_ and the long-term thermodynamic stability of such solid solution. The results of our studies are not limited to the field of nuclear waste management and in a general science aspect could be used to improve computational methods (Blanca-Romero et al., 2014) or the monazite-xenotime geothermometry (Mogilevsky, 2007). We especially highlight a cross-linking, interdisciplinary character of our research, from which the general science community could highly benefit.

## 2. Computational Approach

In the *ab initio*^1^ investigation of the lanthanide phosphate systems we presented here we apply a density functional theory (DFT)-based quantum chemistry approach. For that purpose we utilize Quantum-ESPRESSO simulation package (Giannozzi et al., 2009). We apply the PBESol exchange-correlation functional (Perdew et al., 2008), the plane-wave energy cutoff of 50 Ryd and ultrasoft pseudopotentials to represent the core electrons of the atoms (Vanderbilt, 1990). Following our broad experience on computation of lanthanide phosphates (Blanca-Romero et al., 2014; Beridze et al., 2016) we apply two methods: (1) with 4*f* electrons included into the pseudopotential core and (2) with 4*f* electrons computed explicitly, which we use for DFT+*U* calculations. The DFT+*U* calculations were performed with the Hubbard *U* parameter values computed from first principles using the linear response method of Cococcioni and de Gironcoli (2005). These computational setups were extensively tested and proved to give very good results for monazite-xenotime systems (Blanca-Romero et al., 2014; Beridze et al., 2016).

Besides quantum chemistry methods, force-field-based molecular modeling has been also used in the investigation of lanthanide phosphates. In this approach, the interatomic interactions are described by an analytical function, for instance by the pair interaction potentials such as Buckingham type (Buckingham, 1938). The advantage of this method is that it allows for simulations of systems containing thousands and even millions of atoms, as opposite to more computationally intensive DFT-based methods, which is currently suitable for simulations of up to a few hundred atoms (Jahn and Kowalski, 2014). The force-field methods are used for the large scale simulations of processes such as radiation cascades (Ji et al., 2017c) or computationally intensive computation of statistical distributions, like different possibilities of incorporating doping elements (Huittinen et al., 2017, 2018). For that purpose, there has been some development in the force fields for lanthanide phosphates (Ji et al., 2017c; Jolley et al., 2017). Nevertheless, these methods do not guarantee to deliver information on the level of quantum-chemical methods. Interaction potentials are also not guaranteed to be transferable, even between the system of identical chemical composition like monazite and xenotime.

## 3. Results and Discussion

### 3.1. Structural Data

The first test of a computational method is its ability to reproduce the measured lattice parameters of the computed crystalline solid. The lattice parameters of lanthanide phosphates were measured by different studies and are known for all the *Ln* cations, except Pm (Ni et al., 1995; Clavier et al., 2011). These could be also accurately estimated from the values of ionic radii of *Ln* cations (Shannon, 1976; Ni et al., 1995). The first DFT-based computational studies of structural parameters of lanthanide phosphates were performed for *LaPO*_4_ monazite (Wang et al., 2005). In these studies the applied PW91 exchange-correlation functional (Perdew and Wang, 1992) resulted in slight overestimation of lattice parameters (by ~1% on average). Rustad (2012) used the PBE exchange-correlation functional (Perdew et al., 1996) to compute structural and thermochemical parameters of all lanthanide phosphates and obtained lattice parameters that are also ~1% too large. In similar, extensive studies of *LnPO*_4_ monazite compounds, Feng et al. (2013) applied local spin approximation, but obtained lattice parameters that are far smaller than the experimental values (up to 10% underestimation of volume). These studies show that the structural parameters of lanthanide-phosphates are very sensitive to the applied computational method, especially to the exchange-correlation functional. A correct treatment of strongly correlated 4*f* electrons also plays an important role here.

**Figure 1:**
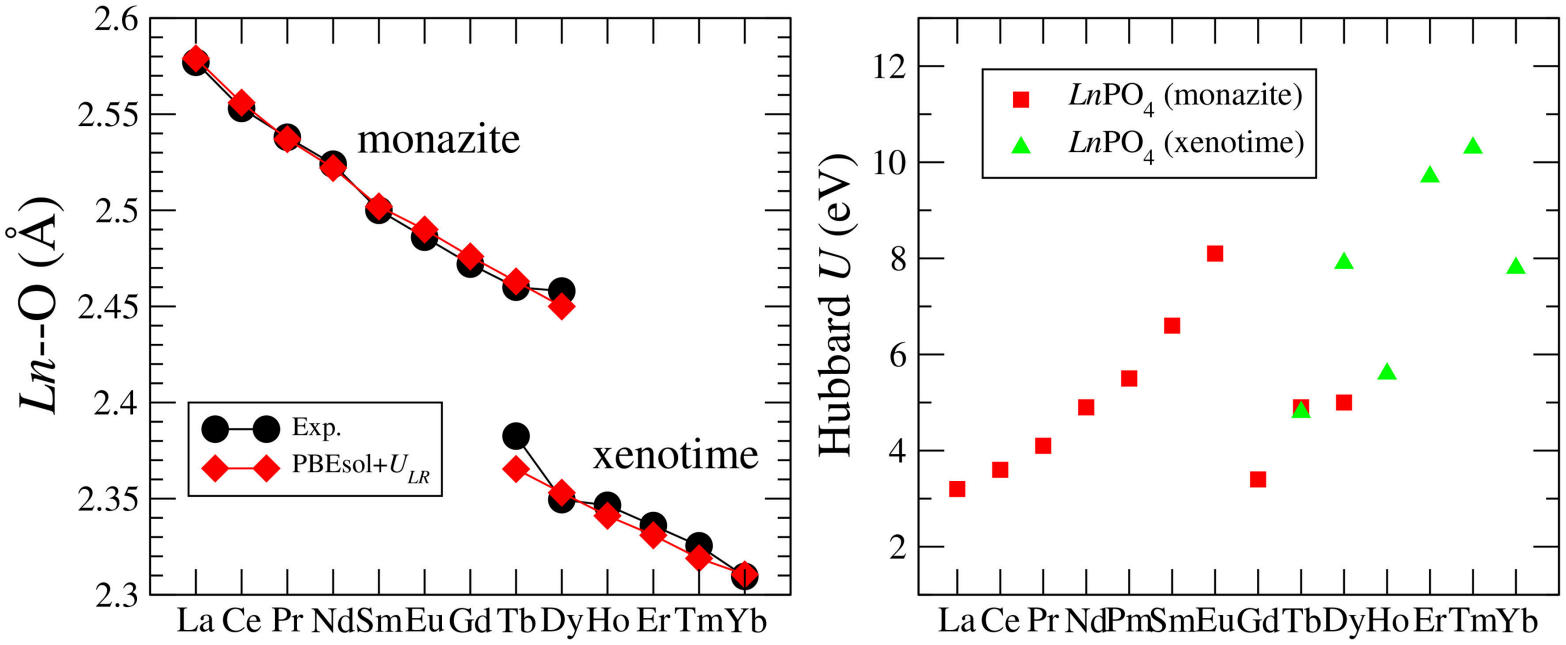
**(Left)** The measured and computed *Ln*-O bond-length in *LnPO*_4_ compounds. **(Right)** The Hubbard *U* parameter computed for *LnPO*_4_ compounds. The data come from Blanca-Romero et al. (2014) and Beridze et al. (2016).

Blanca-Romero et al. (2014) performed extensive tests of the ability of different DFT-based approaches to reproduce the measured lattice parameters and bond-distances of monazite-type lanthanide-phosphates and the related lanthanide-oxides. In that paper, they found that the standard DFT method with explicitly computed 4*f* electrons overestimates the lattice parameters and bond-lengths by up to 3%, consistent with previous studies (Wang et al., 2005; Rustad, 2012). A very good match to the experimental values of structural parameters was obtained with the DFT+*U* method, in which the PBEsol exchange-correlation functional was applied (Perdew et al., 2008) and the Hubbard *U* parameters that represent the strength of the on-site Coulomb repulsion between 4*f* electrons (thus electronic correlations), were derived *ab initio*. In Figure 1 we present the *Ln*-O bond lengths computed with this method vs. the measured data for monazite and xenotime systems. The match is excellent and the key to get such a good result was to compute the Hubbard *U* parameters for each *Ln* case. As demonstrated in Figure 1, this parameter varies a lot for the different *Ln* cations (from ~3 to ~10*eV*). Having an improved description of structures using the PBEsol exchange-correlation functional is also expected, as by recovering the exact charge density limit for slowly varying densities, it improves the description of structural parameters over the widely used PBE functional [see discussion by Perdew et al. (2008)]. Another practice in computation of 4*f* elements is to include the 4*f* electrons into the pseudopotential core and to not compute them explicitly [as done, for instance, by Rustad (2012)]. This is due to the fact that these do not participate in the chemistry (bonding). Blanca-Romero et al. (2014) and Beridze et al. (2016) illustrated this for lanthanide phosphates. They found that such a “*f in the core"* approach results in the formation enthalpies (energies) that are consistent with the ones derived with the DFT+*U* method (see discussion in section 3.2). This method, although not that accurate for the structural parameters as the aforementioned DFT+*U* approach (Beridze et al., 2016), was shown to be accurate for prediction of the thermodynamic and elastic parameters of lanthanide phosphates, which is discussed in the following sections.

### 3.2. Formation Enthalpies

**Figure 2:**
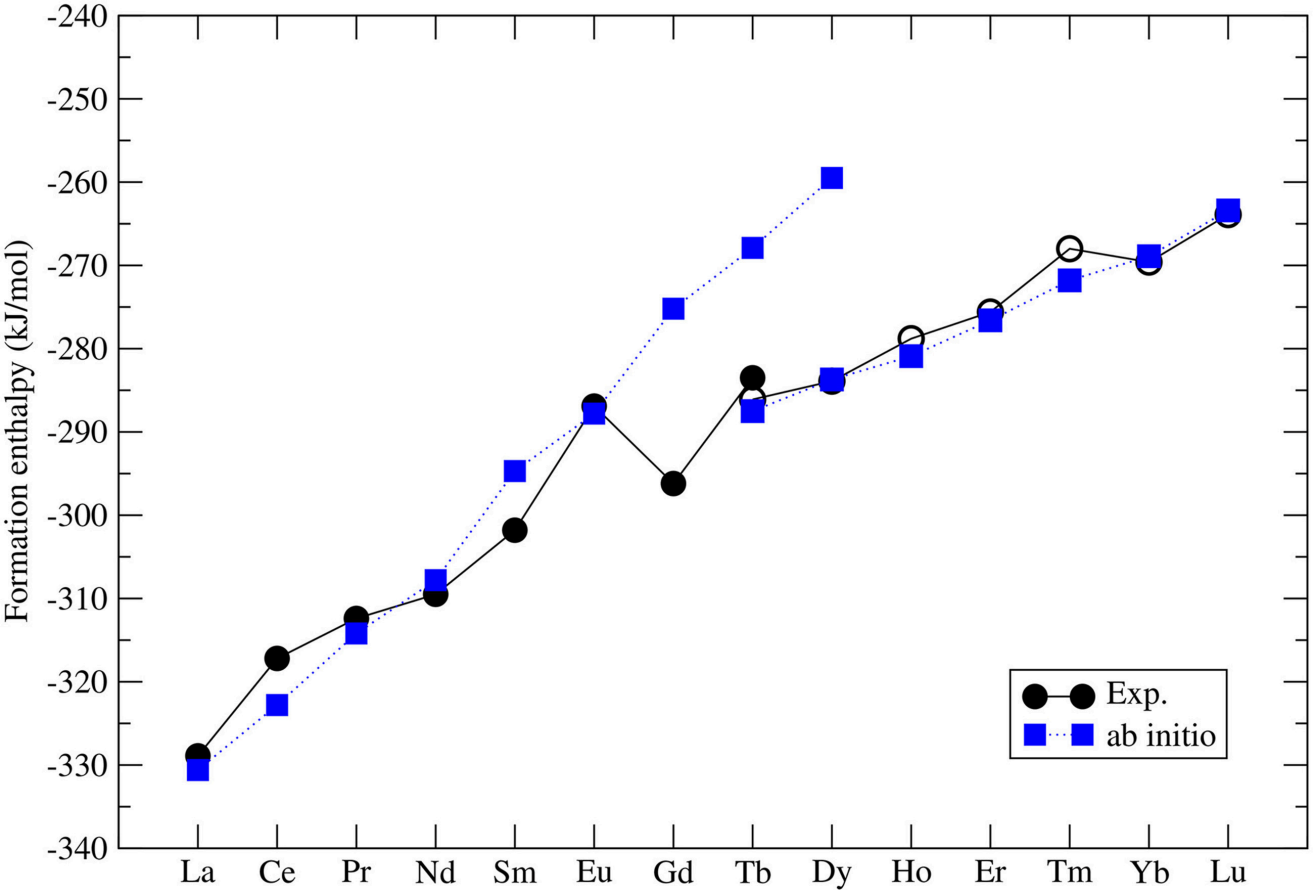
The computed (blue filled squares) and measured (black filled (monazite) and open (xenotime) circles) (Ushakov et al., 2001) formation enthalpy from oxides for *LnPO*_4_ compounds. The following *Ln*_2_*O*_3_ oxides were taken into account: *A*-type (La-Nd), *B*-type (Sm), and *C*-type (Eu-Lu). The data comes from Beridze et al. (2016).

The formation enthalpies of series of monazites and xenotimes have been measured by Ushakov et al. (2001). These were first computed at the DFT level by Rustad (2012). He noticed that there is a systematic offset between the computed and measured values of ~40*kJ*/*mol*, with the computed enthalpies being less exothermic. Blanca-Romero et al. (2014) have shown that this offset is present also in the DFT+*U* calculations and is to a large extent *Ln*-cation independent, which rules out the 4*f* electrons correlations as the underlying reason for it. They attributed this to the overestimation of P-O bond lengths, and thus volumes, of the *LnPO*_4_ and *P*_2_*O*_5_ compounds. Beridze et al. (2016) have found an identical offset for xenotimes. When a constant shift of ~30*kJ*/*mol* is applied to the computed formation enthalpies the measured values are nicely reproduced. The final result for series of *LnPO*_4_ compounds is given in Figure 2. The computed formation enthalpies reproduce well the experimental trend for most of the compounds. The only significant discrepancies are observed for *GdPO*_4_ and *TbPO*_4_, which however is crucial for the discussion of the relative solubilities provided in section 3.7.

**Figure 3:**
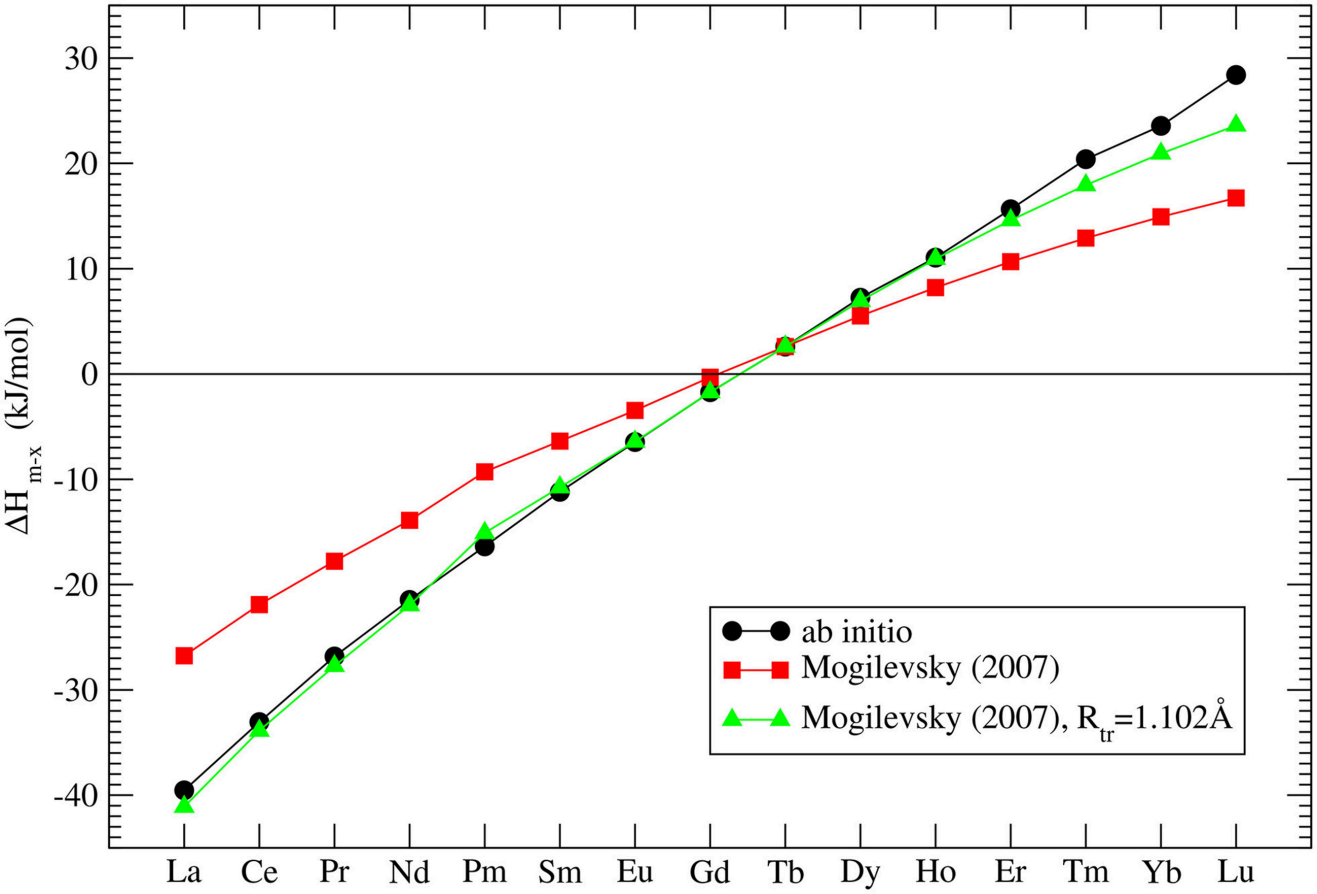
The computed enthalpy difference between monazite and xenotime *LnPO*_4_ compounds. The three data series represent our *ab initio* calculations (black filled circles), the model of Mogilevsky (2007) (red filled squares) and the model of Mogilevsky (2007) (Equations 1, 2) assuming the ionic radius of the 9-fold coordinated transition *Ln* cation is 1.102Å.

The correct prediction of the formation enthalpies of monazite and xenotime phases is essential to properly model the relative solubilities in the monazite-xenotime system. Mogilevsky (2007) in his estimations used an extrapolation of the experimentally measured values for *TbPO*_4_ (Ushakov et al., 2001) - the only system for which formation enthalpies of both phases have been measured. Mogilevsky (2007) extrapolated these values by the following formula:

(1) $$\Delta h_{x}=(R_{tr}-R_{x})/(R_{tr}-R_{Tb})\Delta h_{{\text{TbPO}}_{\text{4}}},$$

(2) $$\Delta h_{m}=(R_{m}-R_{tr})/(R_{tr}-R_{Tb})\Delta h_{{\text{TbPO}}_{\text{4}}},$$

where *R*_*tr*_ is the ionic radius of a hypothetical ion located between Gd and Tb for which energies in the monazite and xenotime structures are identical, *R*_*m, x*_ is the ionic radius of *Ln* cations, which at normal conditions form monazite (m) or xenotime (x), respectively. He assumed that *R*_*tr*_ = 1.0516Å and derived Δ*h*_*m, x*_ which is the enthalpy difference between the standard phase and the other phase (e.g., for *LaPO*_4_ the enthalpy difference between monazite and xenotime phase of *LaPO*_4_). His result is given in Figure 3. In order to check these values we performed *ab initio* calculations of series of monazite and xenotimes counterparts of the existent phases. The advantage of atomistic simulations is that even non existing phases could be modeled (e.g., *LaPO*_4_ xenotime). The result is given in Figure 3. The computed Δ*h*_*m*−*x*_ is significantly different from the estimation of Mogilevsky (2007), with the differences for *LaPO*_4_ of ~20*kJ*/*mol*. We note that our *ab initio* data could be reproduced assuming *R*_*tr*_ = 1.507Å. Having a good match to the experimental values of the measured compounds (see Figure 2) there is no reason to believe that our prediction for the non-existing phases could be that much wrong. We thus assume that the values computed here are correct.

### 3.3. Elastic Parameters

The elastic properties of lanthanide phosphates have been a topic of several experimental studies (Thomä et al., 1974; Harley and Manning, 1978; Nipko et al., 1996; Mogilevsky et al., 2006; Du et al., 2009; Thust et al., 2015). Also on the computational side these have been extensively investigated. Wang et al. (2005), Feng et al. (2013), and Kowalski and Li (2016) computed elastic constants and moduli for a series of monazites. We note that the later study gave the best match to the measured values. The key factor to obtain accurate results has been a good reproduction of structural parameters of the investigated materials. For instance, Kowalski and Li (2016) obtained volumes that are within 1% of the measured values, while in the earlier studies by Feng et al. (2013) the differences were as large as 10%. In the follow-up studies, Kowalski et al. (2017b) computed the variation of the Young's modulus along the *La*_*1-x*_*Eu*_*x*_*PO*_4_ solid solution, which reproduced the experimental trend (Thust et al., 2015) and allowed for better interpretation of these data. Computational methods were also applied to study the pressure effect on the elastic moduli and the relevant elastic anisotropy (Ali et al., 2016; Gomis et al., 2017).

The computed elastic moduli were also used to derive some related thermodynamic parameters. Feng et al. (2013) and Ji et al. (2017b) used the Slack model (Slack, 1973), and derived thermal conductivities of monazite and xenotime compounds, respectively. According to Slack (1973) the thermal conductivity can be estimated as:

(3) $$\kappa =A\frac{\bar{M}\delta \Theta_{D}^{3}}{\gamma^{2}n^{\frac{2}{3}}T},$$

where *A*=3.12·10^−8^*Wmolkg*^−1^*m*^−2^*K*^−3^ is a constant, $\bar{M}$ is the average atomic mass, δ^3^ is the average volume per atom in the system, Θ_*D*_ is the Debye temperature, *n* is the number of atoms per primitive unit cell and γ is the acoustic Grüneisen parameter. Here, using the same approach as by Ji et al. (2017b) we computed the standard thermal conductivity of *LaPO*_4_. The obtained value of room temperature thermal conductivity of 4.0*Wm*^−1^*K*^−1^ is consistent with the measured value of 3.6*Wm*^−1^*K*^−1^ (Du et al., 2009) and the data computed by Feng et al. (2013). The data for *LaPO*_4_ (m) and *LuPO*_4_ (x) are reported in Table 1. Interestingly, both experiment and simulation show significant differences in the thermal conductivity of the monazite and xenotime phases. This comes mainly from the different number of atoms in the primitive cell of both phases, 12 for xenotime and 24 for monazite (see Equation 3).

**Table 1:** The thermal conductivity in (W m^−1^K^−1^) simulated and measured for LaPO_4_ (monazite) and LuPO_4_ (xenotime).

**Compound**	**Calculations**	**Measurements**
*LaPO*_4_	4.0^a^, 4.5^b^	3.6^c^
*LuPO*_4_	11.7^d^	12.0^e^

The references are these of:

a *this work*,

b *Feng et al. (2013)*,

c *Du et al. (2009)*,

d *Ji et al. (2017b)*,

e *Hikichi et al. (1998)*.

**Figure 4:**
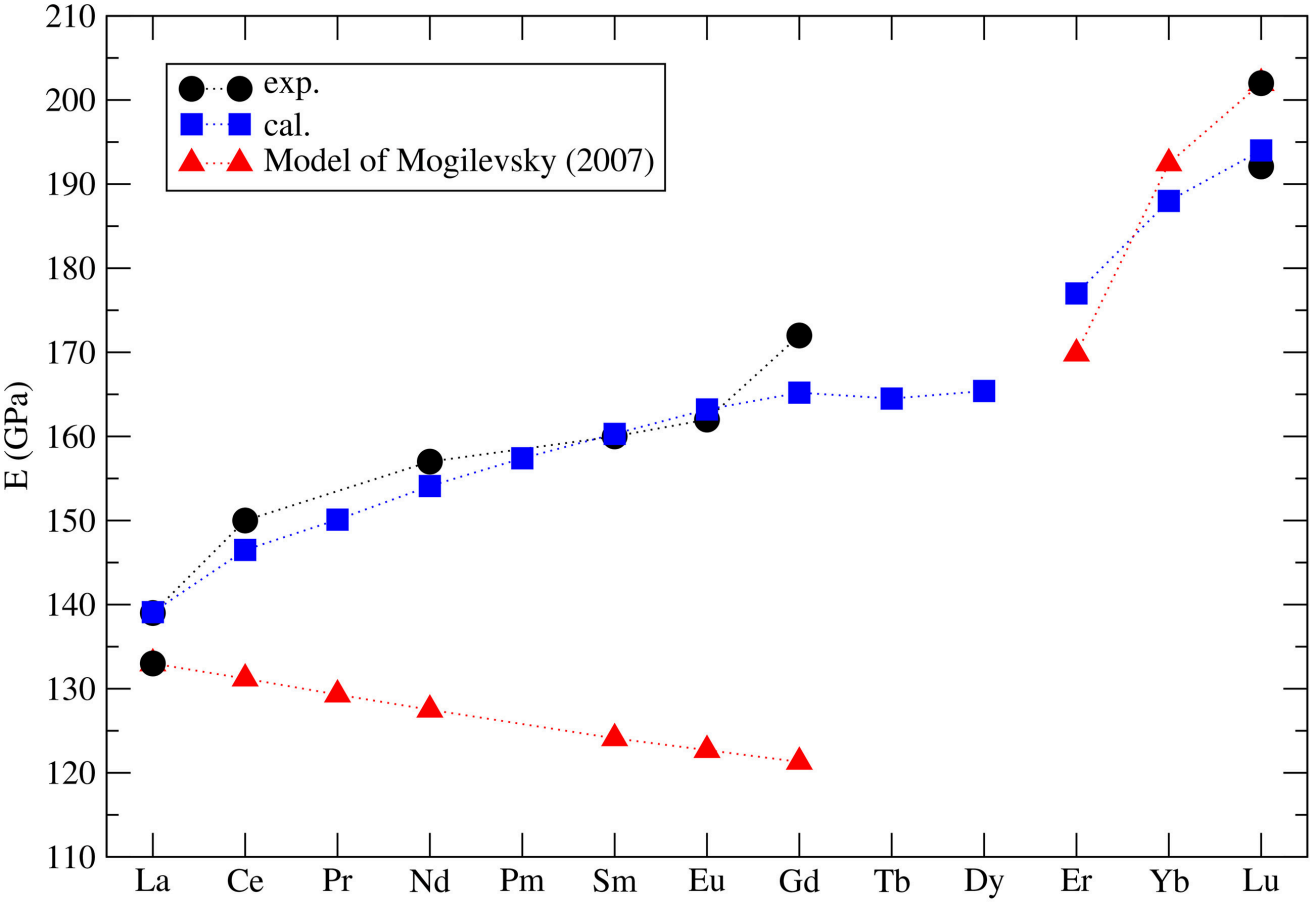
The computed (blue filled squares) (Kowalski and Li, 2016; Ji et al., 2017a) and measured (black filled circles) Young's modulus for selected lanthanide phospahtes monazites and xenotimes. Red triangles represent the model used by Mogilevsky (2007).

Mogilevsky (2007) used the Young's modulus to estimate the excess energy of mixing [the Margules interaction parameter (Prieto, 2009)] in monazite-xenotime solid solution systems. By a combination of fitting to the existing data on solubilities and so computed mixing energies he proposed an accurate model to describe the maximum solubilities and miscibility gap in the monazite-xenotime system. Kowalski and Li (2016) have shown that this Young's modulus-based approximation of elastic strain energy leads to a good description of the *ab initio* Margules interaction parameters. These issues will be discussed in details in sections 3.6 and 3.7. Nevertheless, Mogilevsky (2007) used an approximation for the variation of Young's modulus along lanthanide series. According to his derivation, for monazite compounds the Young's modulus decreases along the series, while the computation of Kowalski and Li (2016) and the aforementioned measurements show an increase (from 140*GPa* for La to ~160*GPa* for Gd). In Figure 4 we present the collection of measured and computed Young's moduli together with the Mogilevsky (2007) approximation. These results are important in the context of estimation of excess enthalpies of mixing and solubilities in the monazite-xenotime system (sections 3.6 and 3.7).

In the next section we will discuss that a careful selection of the computational method, which results in good description of the structural and elastic parameters, is also important to obtain good predictions for thermodynamic parameters such as the heat capacity and the thermal conductivity.

### 3.4. Thermodynamic Parameters

**Figure 5:**
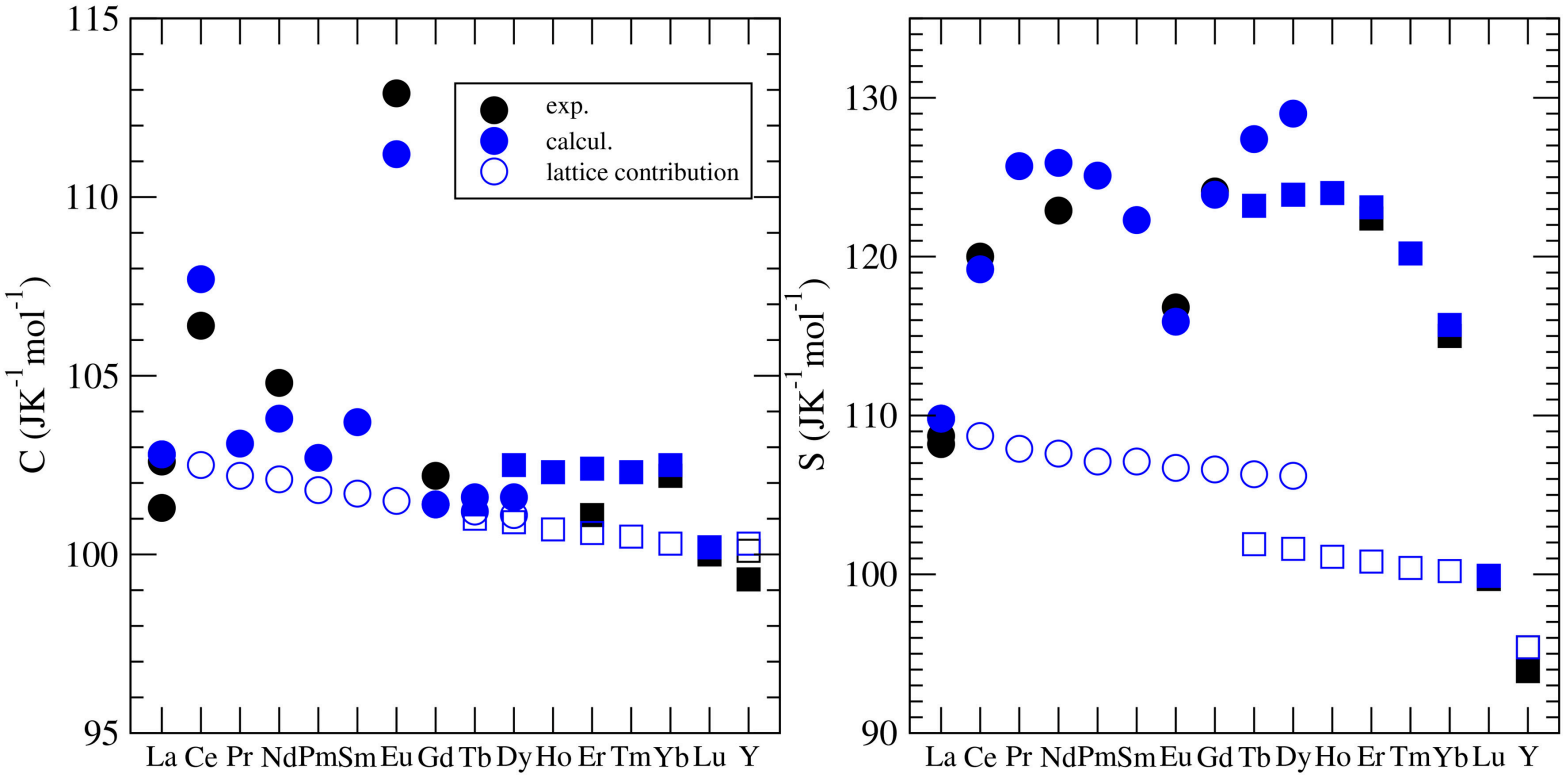
The variation of the standard heat capacity and standard entropy from *LnPO*_4_. The different symbols represent the computed *C*_*v*_ [filled blue cirlces (monazite) and squares (xenotime)] and measured (*C*_*p*_) values [see Kowalski et al. (2015); Ji et al. (2017a)]. The open blue symbols represent the lattice vibration contribution to the heat capacity and entropy. The difference between the filled and open blue circles is due to the thermal excitation of 4*f* electrons (Schottky effect).

Besides the thermal conductivity, the heat capacity is the most widely investigated thermodynamic parameter of lanthanide phosphates. There is a series of experimental papers on measurements of this parameter for different monazite compositions (Thiriet et al., 2004, 2005a; Popa and Konings, 2006; Popa et al., 2006a,b; Gavrichev et al., 2008, 2009; Bauer et al., 2016), including Pu-rich compounds (Thiriet et al., 2005b; Popa et al., 2007a; Benes et al., 2011), and xenotime (Gavrichev et al., 2006, 2010, 2012, 2013; Nikiforova et al., 2012; Gysi et al., 2016). These measurements revealed a quasi-random-like variation of the standard heat capacity and the standard entropy along the lanthanide series. This is illustrated in Figure 5. For instance, the standard values are largest for *EuPO*_4_ and smallest for *GdPO*_4_ with no visible trend. The first systematic computational studies of Feng et al. (2013) were unable to match the experimental data and tried to explain these discrepancies by the large difference between computed constant volume (*C*_*v*_) and measured (*C*_*p*_) heat capacities. In order to shed the light on this variation, Kowalski et al. (2015) and Ji et al. (2017a) performed accurate *ab initio* calculations of the heat capacity of monazites and xenotimes, respectively. They accounted for two effects: (1) the lattice vibration and (2) the thermal excitation of 4*f* electrons, which had been known from experimental studies to significantly influence the heat capacity of monazites [e.g., Thiriet et al. (2005a,b)]. By doing so, Kowalski et al. (2015) were able to accurately reproduce the measured values for all the investigated cases. Using the same computational approach, Kowalski et al. (2017b) were able to explain the low temperature variation of heat capacity in the *La*_1−*x*_*Eu*_*x*_*PO*_4_ monazite-type solid solution system (Thust et al., 2015). By applying a theoretically justified extrapolation, Kowalski et al. (2015) could also account for high temperature (*T* > 800*K*) anharmonic effects in monazites. We note however, that such a procedure was not that successful for xenotime phases, as here the anharmonic effects become important at a much lower temperature of ~400*K* (Ji et al., 2017a). Computational studies of the heat capacity of xenotime by Ji et al. (2017a) allowed also for the verification of the slightly conflicting experimental data sets published recently in the literature (Gavrichev et al., 2010, 2012, 2013; Gysi et al., 2016).

The thermodynamics of lanthanide phosphate solid solutions is discussed in section 3.6.

### 3.5. Incorporation of Radionuclides

Incorporation of actinides into lanthanide phosphates ceramic is an important topic in terms of using these materials as potential nuclear waste forms. It is well known that the trivalent actinides such as Pu, Am and Cm could form monazite phases (e.g., *PuPO*_4_ Thiriet et al., 2005b; Clavier et al., 2011) and that the tetra-valent actinides such as U or Np can be introduced into the monazite lattice jointly with divalent cations [e.g., Ca, Clavier et al. (2011)]. However, an important aspect of the formation of such a solid solution is its homogeneity. In that respect, Huittinen et al. (2017) and Huittinen et al. (2018) investigated incorporation of Eu^3+^ into monazite and Cm^3+^ into monazite and rhabdophane phases, respectively. The latter compound is a hydrated lanthanide-phosphate which is a stable phase at lower temperatures (below *T* ~ 400*K*) (de Kerdaniel et al., 2007; Clavier et al., 2011). By a combination of time-resolved laser fluorescence spectroscopy (TRLFS) and atomistic modeling, Huittinen et al. (2017) have shown that monazite-type solid solutions are homogeneous. This conclusion was reached by explanation of the increased broadening of TRLFS profiles for the *La*_*1-x*_*Gd*_*x*_*PO*_4_ solid solutions by the broad distribution of the dopant-O bond lengths in the measured mixed compounds. In these studies force-field methods were used to describe the interatomic interactions and special quasi-random structures (SQS) (Zunger et al., 1990) were used to model the ideal, homogeneous solid solutions. The same approach was used by Huittinen et al. (2018) to show homogeneous incorporation of *Cm*^3+^ into monazite. On the other hand, rhabdophane-type *La*_1−*x*_*Gd*_*x*_*PO*_4_ solid solutions were found to be very selective in terms of actinides incorporation, with the anhydrous cation site (i.e., not coordinated to *H*_2_*O* molecules) being preferred by the Cm dopant, and the preference increasing toward larger and lighter lanthanides [greater for *LaPO*_4_ · 0.67*H*_2_*O* than *GdPO*_4_ · 0.67*H*_2_*O*, Huittinen et al. (2018)]. This selectivity was confirmed by the computation of the Cm solution (incorporation) energies.

### 3.6. Solid Solutions

Formation and thermodynamic stability of solid solutions of radionuclides-bearing lanthanide phosphates ceramic waste forms is a topic of extensive studies in the context of nuclear waste management (Popa et al., 2007a; Li et al., 2014; Kowalski and Li, 2016; Arinicheva et al., 2017; Hirsch et al., 2017; Neumeier et al., 2017b; Eremin et al., 2019). This is because the information gained allows for the assessment of long term stability of ceramic nuclear waste forms against phase separation. In that aspect there are two streams of research: (1) the investigation of homogeneity of a solid solution discussed in section 3.5 and (2) the investigation of its long-term thermodynamic stability. The second problem is a key issue for nuclear waste disposal because any desired waste form should be stable against formation of the miscibility gap. It was shown experimentally (Popa et al., 2007b) and by *ab initio* simulations (Li et al., 2014) that monazite-type solid solutions are highly regular. The excess enthalpy of mixing, *H*^*E*^, of a (*A*_1−*x*_*B*_*x*_*PO*_4_) could be described by a simple equation (Popa et al., 2007b):

(4) $$H^{E}=Wx(1-x),$$

where *W* is a Margules interaction parameter (Prieto, 2009). A solid solution is stable against formation of a miscibility gap if *W* < 2*RT*, where *R* is the gas constant. It is thus of interest to nuclear waste management strategies to characterize *W* for the considered solid solutions, especially for the mixture of actinides with the host matrix cations like Pu with La in a *La*_*1-x*_*Pu*_*x*_*PO*_4_ solid solution.

The first systematic *ab initio* calculations of *W* parameters for monazite-type solid solutions were performed by Li et al. (2014). They found that for the *La*_1−*x*_*Ln*_*x*_*PO*_4_ solid solutions, *W* = 0.618(Δ*V*(cm^3^/mol))^2^, where Δ*V* is the difference in the volume of solid solution endmembers. The obtained results suggest thermodynamic instability, characterized with *W* > 5*kJ*/*mol* at ambient conditions, of various solid solutions [e.g., (*La*_1−*x*_*Gd*_*x*_*PO*_4_)]. In follow-up studies, Kowalski and Li (2016) explained the quadratic dependence of *W* parameter on the volumes difference by a strain energy-based model, in which

(5) $$W=\frac{E}{6V}{\left(\Delta V\right)}^{2},$$

where *E* is the Young's modulus and *V* is the volume. These studies show that Δ*V* is an important parameter that determines the value of the *W* parameter.

Neumeier et al. (2017b) compared the derived *ab initio* *W* parameters with the calorimetric measurements of *La*_1−*x*_*Ln*_*x*_*PO*_4_ (*Ln* = *Eu, Gd*) solid solutions. The measured values are smaller than the computed ones. The reason for this discrepancy is the difference in the value of measured and computed Δ*V*. These are summarized in Table 2. When Neumeier et al. (2017b) used the measured Δ*V* values and rescaled the *ab initio* computed values according to Equation 5, namely:

(6) $$W={\left(\frac{\Delta V_{exp}}{\Delta V_{comp.}}\right)}^{2}W_{computed},$$

as provided in Table 2, they obtained a good match to the measured values.

**Table 2:** The Margules interaction parameters *W* and the difference in volumes of endmembers (Δ*V*) for La_1−*x*_Ln_*x*_PO_4_ (Ln = Eu, Gd, Pu) monazite-type solid solutions.

**Compound**	**Δ*V*_*computed*_**	**Δ*V*_*measured*_**	***W_ab initio_^a^***	***W_rescalled_^b^***	***W_measured_^c^***
*La*_*1−*x**_*Eu*_**x**_*PO*_4_	7.6	5.2	13.4	6.2	2.5 ± 2.6
*La*_*1−*x**_*Gd*_**x**_*PO*_4_	8.4	6.1	16.5	8.6	11.4 ± 3.1
*La*_*1−*x**_*Pu*_**x**_*PO*_4_	4.3	2.9	4.0	1.9	

The provided W parameters come from:

a *Li et al. (2014)*,

b *Kowalski and Li (2016)*,

c *Neumeier et al. (2017b)*.

The most important outcome of these studies is their implication for immobilization of actinides, for instance Pu. The obtained Margules interaction parameter for *La*_1−*x*_*Pu*_*x*_*PO*_4_ solid solution case is *W* = 1.8*kJ*/*mol* (Table 2), thus indicating thermodynamic stability of this solid solution against formation of a miscibility gap (as *W* is < 5*kJ*/*mol*). On the other hand, although synthesis of the pure *PuPO*_4_ monazite has been successful (Thiriet et al., 2005b; Clavier et al., 2011), the experimental studies suggest a maximum content of Pu in *La*_1−*x*_*Pu*_*x*_*PO*_4_ solid solution at *x* ~ 0.15 (Popa et al., 2007a; Arinicheva et al., 2017). In particular, recent experimental investigation by Arinicheva et al. (2017) indicates formation of Pu-oxides (*PuO*_2_) for *x* > 0.15. In order to understand this contradiction, we computed the formation enthalpy of *La*_1−*x*_*Pu*_*x*_*PO*_4_ solid solution from oxides. We consider the following reaction:

(7) $$\frac{1}{2}(1-x){\text{La}}_{\text{2}}{\text{O}}_{\text{3}}\text{+}\frac{1}{2}x{\text{Pu}}_{\text{2}}{\text{O}}_{\text{3}}\text{+}\frac{\text{1}}{\text{2}}{\text{P}}_{\text{2}}{\text{O}}_{\text{5}}\to {\text{La}}_{1-x}{\text{Pu}}_{x}{\text{PO}}_{\text{4}}$$

**Figure 6:**
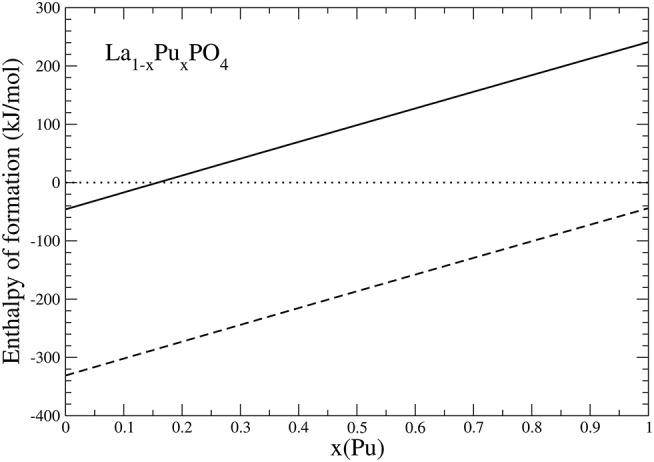
The formation enthalpy from oxides of La_1−x_Pu_x_PO_4_ solid solution compounds. The lines represent results taking Pu_2_O_3_ (dashed) and PuO_2_ (solid) as a reference.

Because *PuO*_2_ is the most stable Pu-oxide at ambient conditions, we also account for the enthalpy of oxidation of *Pu*_2_*O*_3_ of 285*kJ*/*mol* (Konings et al., 2014). The result is given in Figure 6. Interestingly, the formation enthalpy becomes positive, and the solid solution becomes unstable against formation of *PuO*_2_ for *x* ~ 0.15. We thus conclude that the experimentally observed maximum load of Pu in *LaPO*_4_ results from the positive formation enthalpies, and thus thermodynamic instability of La_1−x_Pu_x_PO_4_ compounds for *x* > 0.15 ^2^.

### 3.7. Solubilities in Monazite-Xenotime System

The thermodynamic parameters of mixing, such as the *W* parameter, are very important for assessment of thermodynamic stability of monazite-type nuclear waste forms. Therefore, it is of high importance to validate the model presented in section 3.6 against all the available data. Interestingly, the Margules interaction parameters are equally important for the description of the miscibility gap, and the maximum solubilities, in the naturally occurring monazite-xenotime systems. The monazite-xenotime system has been proposed as a potential geothermometer and some relevant data on solubilities have been measured (Andrehs and Heinrich, 1998; Mogilevsky, 2007). Monazite-xenotime system shows a large miscibility gap, which is associated with a large difference between the formation enthalpies of monazite and xenotime (Figure 3). Nevertheless, the measured maximum solubilities can be good indicators of temperature and this phenomenon have been investigated “*in situ*” experimentally (Andrehs and Heinrich, 1998), and by thermodynamic modeling (Mogilevsky, 2007). In order to model the maximum solubility of one phase in another we have to know the Margules interaction parameters *W* and the difference in the formation enthalpies between monazite and xenotime phases. The first is discussed in section 3.6 and as we concluded there, we have a good model for it. The latter is discussed in section 3.2.

**Figure 7:**
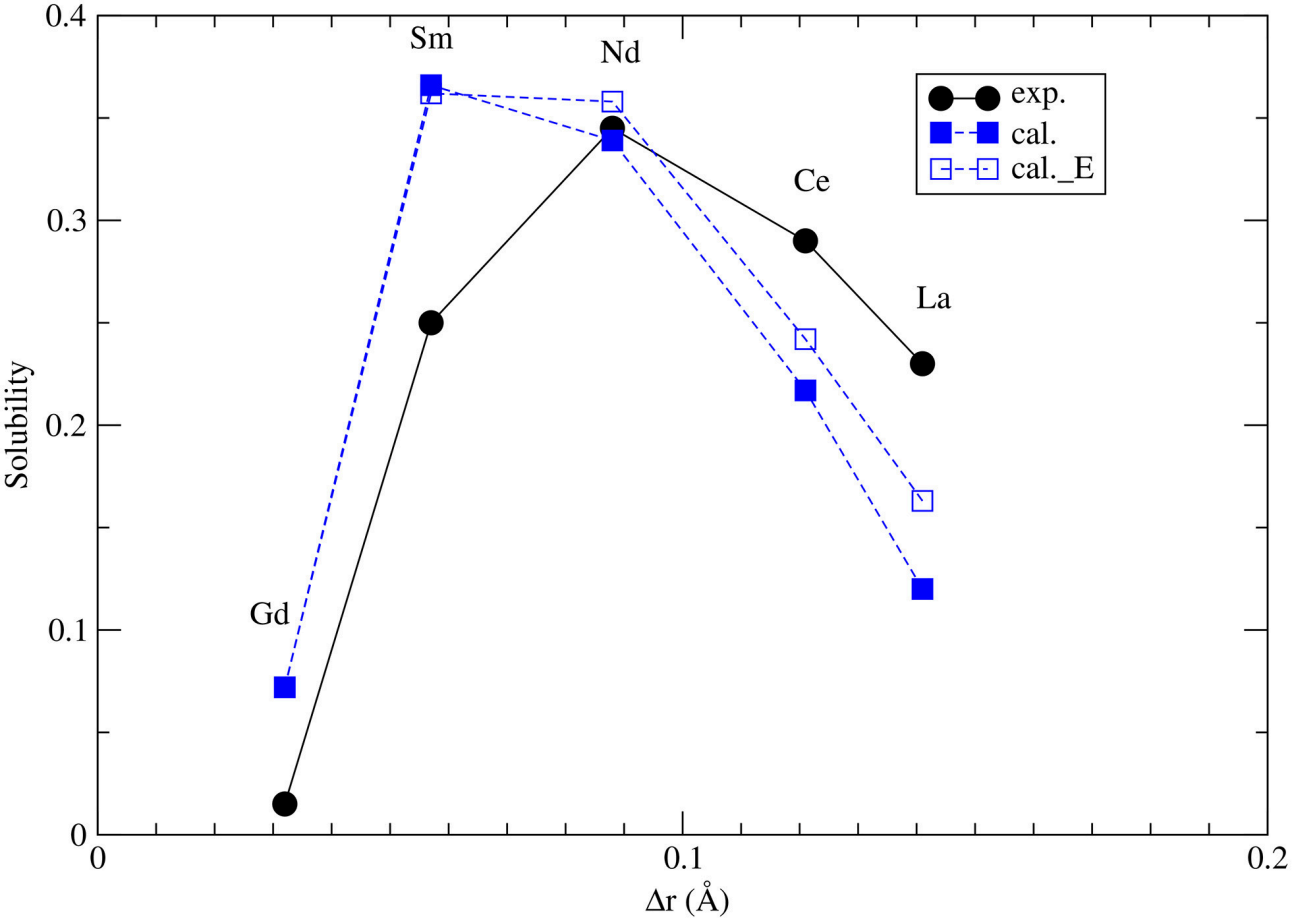
The computed maximum solubility of Y-xenotime in monazite at *T* = 1473*K*. Different sets represent our *ab initio* prediction (filled blue squares), prediction using Equation 5 [open blue squares, Kowalski and Li (2016)] and measurements [filled black circles, Andrehs and Heinrich (1998); Mogilevsky (2007)]. Δ*r* is the difference in ionic radius of *Ln* and *Y* cations.

The maximum solubilities of xenotime in monazite (*x*_1_) and monazite in xenotime (*x*_2_) are obtained by solving self-consistently two equations (Mogilevsky, 2007):

(8) $$\Omega_{m}{\left(1-x_{1}\right)}^{2}+RT\ln(x_{1}/(1-x_{2}))=\Delta h_{x}+\Omega_{x}x_{2}^{2},$$

(9) $$\Omega_{x}{\left(1-x_{2}\right)}^{2}+RT\ln(x_{2}/(1-x_{1}))=\Delta h_{m}+\Omega_{m}x_{1}^{2},$$

where Ω_*x,m*_ = −Δ*h*_*m,x*_ + *W*_*m,x*_. Δ*h*_*m,x*_ is given by Equations 1, 2 (see also section 3.2) and *W*_*m, x*_ are the Margules interaction parameters for monazite (m)- and xenotime (x)-type solid solutions. The self-consistent solution of Equations 8 and 9 can be easily done in a numerical way. The resulted maximum solubilities for the selected solid solution compositions at *T* = 1453*K* are given in Figure 7. The solubility of xenotime in monazite is much larger than monazite in xenotime (Mogilevsky, 2007), so here we focus on the former phenomenon. For the solubility of Y-xenotime (*YPO*_4_) in monazite there is a maximum at Sm - Nd. We note that the match to *in situ* data (Andrehs and Heinrich, 1998) is not perfect and is not that good as the empirically-based thermodynamic modeling of Mogilevsky (2007), but the trend is well described on the qualitative level. The main discrepancy comes from the Δ*H*_*m*−*x*_, which we have reasons to think are quite accurate by the *ab initio* calculations. Therefore, more testing against larger data sets would be required to work out and validate a more accurate model for the maximum solubilities in the monazite-xenotime system. This example also shows the importance of correct thermodynamic modeling of monazite-xenotime system, which could be performed and validated by a joint atomistic modeling and experimental effort.

## 4. Conclusions

In this contribution we presented recent atomistic modeling contribution to the research on lanthanide phosphates, focusing on the studies performed at Forschungszentrum Jülich. We have shown that on many occasions joint atomistic modeling and experimental work resulted in the enhanced description of the investigated materials. Examples of such joint studies are investigations of homogeneity and thermodynamic parameters of solid solutions and incorporation of actinides into lanthanide phosphate ceramics. With the improved understanding and description of the 4*f* electrons, achieved with the parameter free DFT+*U* approach, we were able to obtain very accurate description of the local *Ln* − *O* bonding environments and the formation enthalpies. This allowed us to obtain better estimates of the relative solubilities in the monazite-xenotime system, elastic and thermodynamic parameters for pure phases and solid solutions. The joint modeling and experimental investigation of the excess enthalpies of mixing in monazite-type solid solutions provided solid arguments for the long-term thermodynamic stability of the plutonium-lanthanum monazite-type ceramic waste form. With respect to our previously published studies, the novel contribution discussed here is the investigation of incorporation of Pu into *LaPO*_4_ ceramics, which shows that the experimentally observed maximum load limit for *La*_*1-x*_*Pu*_*x*_*PO*_4_ is governed by the formation enthalpy from oxides, which becomes positive at ~*x* = 0.15. This value is consistent with the experimental observations. In addition, we reinvestigated the thermodynamics of monazite-xenotime system. We provide accurate values of the enthalpy difference between monazite and xenotime phases and estimates of maximal solubilities computed from the best available *ab initio* data. These results are of great importance for monazite/xenotime-based geothermometry. However, the obtained slight discrepancies between theoretical prediction and the available *in situ* data show a need for more in-depth experimental and theoretical studies of solubilities in the monazite-xenotime system.

The assortment of results provided here shows that atomistic modeling is a valuable research tool for the investigation of lanthanide phosphates. The best results have been obtained by a joint computational and experimental approach, or at least by extensive testing and comparison to the available experimental data. With the steady increase in the availability of computational power we expect that atomistic modeling research will be applied to tackle more complex problems, such as dissolution or corrosion kinetics, an important aspects of nuclear waste performance.

## Data Availability

All datasets generated for this study are included in the manuscript and/or the supplementary files.

## Author Contributions

YJ and PMK contributed equally to computing the data, analysis and editing the manuscript. PK, NH, and YA contributed experimental support and expertise. VV contributed to the computation of thermodynamic parameters of solid solutions. NM contributed to the computation of thermal conductivity and force-field simulations. SN and DB contributed expertise on ceramic waste forms.

### Conflict of Interest Statement

The authors declare that the research was conducted in the absence of any commercial or financial relationships that could be construed as a potential conflict of interest.

## Footnotes

^1^In this contribution we call DFT methods an *ab initio* approach as the exchange-correlation functionals utilized in our studies were designed based on pure-theoretical considerations.

^2^We notice that in order to correctly quantify the thermodynamics of this process, the free energies should be considered. However, the consideration of the entropy terms (main contributor to the difference between the enthalpies and the free energies of the reaction 7) in the reaction 7 would slightly increase the threshold *x* value but on qualitative level, the outlined picture will not change.
